# Bioassay Using the DR-EcoScreen System to Measure Dioxin-Related Compounds in Serum Samples from Individuals Exposed to Dioxins Originating from Agent Orange in Vietnam

**DOI:** 10.3390/toxics13060513

**Published:** 2025-06-19

**Authors:** Masafumi Nakamura, Ryo Matsuda, Hoa Thi Vu, Tai Pham-The, Thao Ngoc Pham, Tomoya Takiguchi, Hisao Nishijo, Muneko Nishijo

**Affiliations:** 1 Hiyoshi Corporation, Omihachiman 523-8555, Japan; m.nakamura@hiyoshi-es.co.jp (M.N.); r.matsuda@hiyoshi-es.co.jp (R.M.); 2 Department of Military Hygiene, Vietnam Military Medical University, Hanoi 12108, Vietnam; vuhoa5593hvqy@gmail.com; 3 Biomedical and Pharmaceutical Research Centre, Vietnam Military Medical University, Hanoi 12108, Vietnam; taithuy@kanazawa-med.ac.jp; 4 Department of Functional Diagnosis, Military Hospital 103, Vietnam Military Medical University, Hanoi 12108, Vietnam; phamngocthaovmmu@gmail.com; 5 Epidemiology and Public Health, Kanazawa Medical University, Uchinada 920-0293, Japan; ttakiguc@kanazawa-med.ac.jp; 6 Department of Sport and Health Sciences, Faculty of Human Sciences, University of East Asia, Shimonoseki 751-8503, Japan; nishijo@toua-u.ac.jp

**Keywords:** dioxins, bioassay, biological equivalency, the World Health Organization toxic equivalent, serum samples, Vietnamese men

## Abstract

A bioassay for dioxin analysis of human samples has the advantages of cost effectiveness and requiring only a small sample volume. Using a DR-EcoScreen bioassay, we measured the biological equivalency (BEQ) levels in serum samples from 32 men exposed to dioxins in Bien Hoa and 32 unexposed men in Hanoi, Vietnam. For the Bien Hoa men, the World Health Organization toxic equivalent (WHO-TEQ) levels of dioxins had already been measured by instrumental analysis. The difference in fat-based BEQ levels between exposed and unexposed men was greater than for crude BEQ levels, with a strong correlation between fat-based BEQ and WHO-TEQ levels. The fat-based BEQ levels in Bien Hoa men with longer residency but lower WHO-TEQ levels were significantly higher than those in unexposed men and Bien Hoa men with shorter residency but higher WHO-TEQs, suggesting that fat-based BEQ may be an effective marker of dioxin-like activity. Additionally, comparisons of 2,3,7,8-tetrachlorodibenzo-p-dioxin (TCDD) and TEQs between shorter- and longer-residency groups indicated that higher levels of polychlorinated dibenzo-p-dioxins (PCDDs), particularly TCDD, contribute to increased BEQ levels. Taken together, the DR-EcoScreen bioassay may be useful to analyze dioxin-like activity associated with WHO-TEQs of men in a dioxin contamination hotspot originating from Agent Orange in Vietnam.

## 1. Introduction

During the Vietnam War, large quantities of herbicides containing polychlorinated dibenzo-p-dioxins and dibenzo-furans (PCDD/Fs) were sprayed in South Vietnam. In the areas around former US airbases in Da Nang, Phu Cat, and Bien Hoa, where herbicides including Agent Orange were stored and prepared for the “Ranch Hand Operation,” PCDD/Fs’ concentrations in the environment and humans have remained elevated even after 40 years [1,2,3,4]. In particular, the Bien Hoa airbase is the largest exposed area to dioxins, specifically 2,3,7,8-tetrachlorodibenzo-p-dioxin (TCDD), originating from Agent Orange [5,6].

PCDD/Fs are lipophilic and can accumulate in the adipose tissue of organisms. Therefore, breast milk, which is rich in milk fat, is a good sample for dioxin analysis. We measured dioxin levels in a total of 861 breast milk samples, including samples from areas around Da Nang and Bien Hoa airbases, using gas chromatography–high-resolution mass spectrometry (GC–HRMS). The dioxin levels in the breast milk in Da Nang and Bien Hoa were found to contain 2–3 times higher levels of the PCDD/Fs congeners, including TCDD, compared with breast milk samples from unsprayed areas [7]. Blood or serum samples are also commonly used specimens to measure dioxin levels in residents and workers exposed to dioxins from environmental and occupational sources [8,9,10,11,12,13,14,15,16,17,18,19]. Van Luong et al. (2018) reported that serum dioxin levels measured by instrumental methods using GC–HRMS were 4–5 times higher in men living around Phu Cat and Bien Hoa airbases compared with those living in an unsprayed area in northern Vietnam [20]. Van Manh et al. (2021) also measured serum dioxin levels in military workers in Bien Hoa airbase using GC–HRMS and reported that TCDD concentrations were two and five times higher than those in workers in Da Nang and Phu Cat airbases, respectively [21]. For fathers from the Bien Hoa birth cohort recruited around the Bien Hoa airbase in 2015, we quantified dioxin congeners in blood by instrument analysis and reported that high levels of PCDD/Fs, particularly TCDD, were associated with alterations in regional brain volume, as analyzed by MRI imaging analysis [22].

However, dioxin analysis of human samples using instrumental methods has disadvantages, such as the large sample volume required to detect the low concentrations of target compound and the high cost and labor intensiveness of investigating a large number of samples in epidemiological studies. By contrast, a bioassay may be more rapid and cost-effective, less labor-intensive, and require a lower sample volume [23,24]. A bioassay could also be a comprehensive evaluation method for dioxin-related compounds, such as brominated dioxins that react to the aryl hydrocarbon receptor (AhR) [25]. Initially, a chemically activated luciferase gene expression (CALUX) bioassay using the mouse hepatoma cell line H1L6.1c3 was developed [26], which was later improved by replacement with a more sensitive cell line, such as H1L7.5c1, to decrease the detection limit of the bioassay system [27]. Using the CALUX bioassay with the H1L7.5c1 cell line, serum samples from adolescents of the second Flemish Environment and Health Study (FLEHS II) were analyzed. The results showed that the detection rates of the biological equivalency (BEQ) levels of PCDD/Fs and dioxin-like polychlorinated biphenyls (dl-PCBs) were higher than those in the CALUX bioassay with the H1L6.1c3 cell line, although the BEQ levels were nearly two times higher than the World Health Organization toxic equivalent (WHO-TEQ) levels of a pooled sample [28].

In Japan, a sensitive bioassay using a mouse hepatoma Hepa1c1c7 cell line, designated the DR-EcoScreen bioassay, was developed, which required only a trace amount for sample and offered sufficiently high recovery to be used for the assessment of dioxin exposure [29,30,31]. Matsuda et al. (2019) confirmed the validity of DR-EcoScreen for analyzing dioxins and optimized the assay using a sample volume of only 0.5 mL of sera or milk and decreased background dioxin levels using a silica gel sulfate column for the cleanup process [32]. Their optimized DR-EcoScreen bioassay was developed for application in health impact studies of exposure to background levels of dioxins and dioxin-like compounds among the general population in Japan.

In the present study, we aimed to measure serum BEQ levels using the DR-EcoScreen bioassay in Vietnamese men who are fathers of our birth cohorts in dioxin-exposed Bien Hoa and unexposed Hanoi to investigate the differences in BEQ levels, corrected and uncorrected by fat content (because of the low fat content in the 0.5-mL serum samples), among residentially categorized groups. For the Bien Hoa fathers, the levels of 17 different 2,3,7,8-substituted dioxin congeners had been measured by GC–HRMS, and the TEQ levels had been calculated with reference to the WHO 2005 TEQ factors (WHO-TEFs) [33] in our previous study [12]. Correlations between the serum BEQ levels in the current analysis and the blood levels of TCDD and TEQs of PCDD/Fs and PCDD/Fs/dl-PCBs previously measured by instrumental methods were analyzed to confirm the applicability of the DR-EcoScreen bioassay in populations highly exposed to dioxins, with a significant contribution of TCDD, originating from Agent Orange in Vietnam.

## 2. Materials and Methods

### 2.1. Study Subjects

In 2015, we recruited infant-and-mother pairs at the time of birth residing nearby Bien Hoa airbase in Dong Nai prefecture, Vietnam (Bien Hoa birth cohort 2015), and found high levels of PCDD/Fs congeners including TCDD in their maternal breast milk [7]. We also recruited infant-and-mother pairs in a herbicide-sprayed area in Hanoi Vietnam, in 2014, and measured dioxin levels of maternal breast milk (Hanoi birth cohort) [7].

In the present study, a total of 64 men, including 32 men who were fathers of the Bien Hoa birth cohort 2015 and 32 men of the Hanoi birth cohort and who had participated in the brain MRI imaging studies as cases and controls [22,34], were enrolled to measure serum BEQ levels by the DR-EcoScreen bioassay. The participant rate of men in Bien Hoa was 58.2% of 55 fathers from the Bien Hoa cohort [22] who were invited for a blood examination of the 1st step of the MRI imaging study, while from the Hanoi cohort, we invited 32 men whose age and educational levels are similar to those of cases in Bien Hoa as controls.

In our previous study, the blood dioxin levels of Bien Hoa fathers, as measured by instrumental analysis, were higher among those who worked at the Bien Hoa airbase than in those with long residency in Bien Hoa [22]. However, a significant alteration in brain regional volume was found among men who had lived in Bien Hoa since birth [34], suggesting the hypothesis that longer residency (dioxin exposure during childhood) may influence AhR activity independently of blood dioxin levels. Therefore, the subjects from Bien Hoa were divided into two groups with 30 years as the cut-off value (which was the 75th percentile value of their length of residency) and their characteristics are shown in Table 1.

The average age was significantly younger among Bien Hoa men with shorter residency (<30 years), but similar among Bien Hoa men with longer residency (≥30 years) compared with unexposed men in Hanoi. With reference to the length of residency and age, men with shorter residency seemed to have moved to Bien Hoa in adulthood, whereas men with longer residency may have lived in Bien Hoa for most of their life. Although the body mass index (BMI) was significantly higher among men with longer residency, serum cholesterol levels were significantly lower in both exposed groups with shorter and longer residency compared with unexposed men.

Written informed consent was obtained from all men according to a process reviewed and approved by the Health Departments of Bien Hoa City and Dong Nai prefectures. The institutional ethics board for medical and health research involving human subjects at Kanazawa Medical University (No. I-424) and Hospital 103 of Vietnam Medical University (No. 107/CNChT-HDDD, 18 November 2022) approved the study design.

### 2.2. Bioassay of Dioxin-like Activity

#### 2.2.1. Analytical Procedure

**Figure 1:**
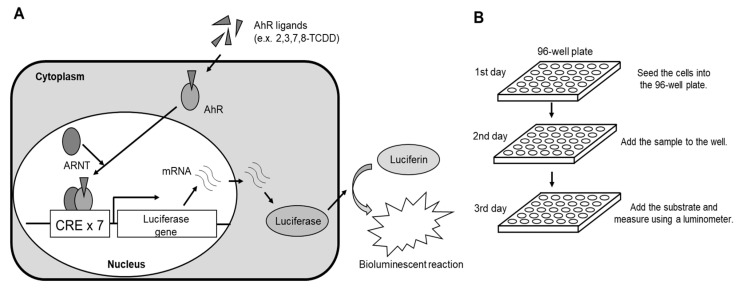
Summary of luciferase reporter gene assay using DR-EcoScreen cells ((**A**): scheme of luciferase induction; (**B**): a rapid and simple procedure).

The BEQ levels in serum samples were measured using the DR-EcoScreen bioassay, the principle (a) and procedure (b) of which are shown in Figure 1. The scheme (Figure 1A) illustrates luciferase induction by a transfected AhR-responsive luciferase reporter gene in DR-EcoScreen cells.

On day 1 of the procedure, DR-EcoScreen cells were prepared at a concentration of 100,000 cells/mL and seeded into a 96-well microplate. The plate was incubated in a humidified atmosphere containing 5% CO2 at 37 °C. On day 2, the cell culture medium (α-MEM supplemented with 5% fetal bovine serum, 2% Pen/Strep antibiotics, and glutamine) was prepared. Test samples (after extraction and cleanup) or standard substances were mixed to achieve a DMSO concentration of 15%, and 10 µL of this mixture was added to each well. The final DMSO concentration in the medium was adjusted to 1.5% [32]. The cells were then exposed to the mixture for 20–24 h. On day 3, 40 µL of Steady-Glo™ reagents (Promega) were added to the exposed cells, and the plate was shaken at room temperature for 2 min. Luciferase activity was subsequently measured using a luminometer (NIVO™, Perkin Elmer, MA, USA) (Figure 1B).

Fat extraction from serum was performed following the method described by Merlo et al. (2013) [35]. Briefly, 1 mL of serum was used for the bioassay. First, 1 mL of isopropanol was added to the sample, and the mixture was vortexed for 1 min. Subsequently, 1 mL of 3% diethyl ether in hexane was added, and the mixture was vortexed for 2 min, followed by centrifugation at 800× *g* for 5 min. The supernatant was collected and this process was repeated three times to extract fat. The extracted solvent was dried under nitrogen, and the fat content (mg) was weighed. The extract was further purified using a 55% sulfuric acid column and finally dissolved in 8 µL of DMSO.

The concentrations of dioxin-related compounds were calculated as TCDD toxic equivalents using 2,3,7,8-TCDD standard curves. The results were expressed as pg-BEQ per g-fat content (fat-based BEQ) and pg-BEQ per g-sample wet weight (assuming 1 mL of sample was equivalent to 1 g of wet weight) (crude BEQ).

#### 2.2.2. Analytical Sensitivity, Recovery, and Reproducibility

**Figure 2:**
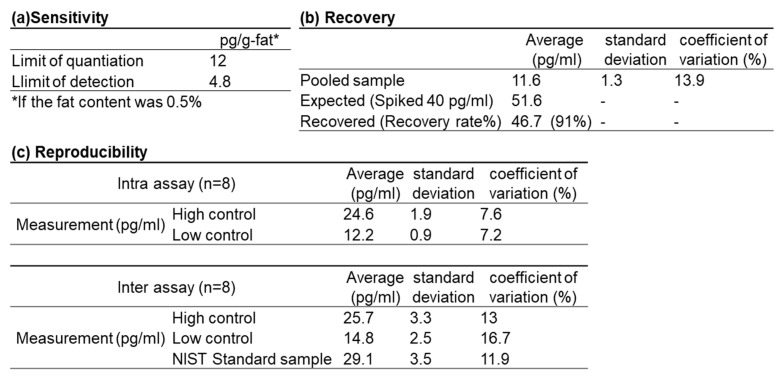
Analytical sensitivity (**a**), recovery (**b**) and reproducibility of inter/intra assay (**c**). (**a**): The limit of quantification and detection, (**b**): the recovery rate performed by spiking the samples with 40 pg/mL of 2,3,7,8-TCDD. (**c**): Coefficients of variation (C.V.) for intra and inter-assay variability when high and low controls and NIST standard sample were measured.

To demonstrate the sensitivity of this assay, the limit of detection (LOD) per 1 mL and the limit of quantification (LOQ) were determined. The LOD was defined as the t-value at degrees of freedom (n = 6) and significance level (α = 0.05) multiplied by twice the standard deviation, while the LOQ was calculated as 10 times the standard deviation (n = 7) of the blank spiked with the standard substance. These values are shown in Figure 2a. When the fat content was 0.5%, the LOD was estimated at 4.8 pg/g-fat, and the LOQ at 12.0 pg/g-fat.

Recovery was tested by spiking the sample with 40 pg/mL of 2,3,7,8-TCDD. The recovery rate was calculated as the percentage (%) of the mean measured values of spiked samples divided by the mean expected values. The recovery rate was 91% (Figure 2b).

Figure 2c shows the coefficients of variation (C.V.) for intra-assay and inter-assay variability, measured for control samples at high and low concentrations as well as for the standard sample. Standard Reference Material 1957 (National Institute of Standards and Technology; NIST, Gaithersburg, MD, USA) was used as the standard sample. The C.V. values were all below 20%, indicating good reproducibility for the DR-EcoScreen assay.

### 2.3. Instrument Analysis of Whole Blood Samples of Men Exposed to Dioxins in Bien Hoa

In our previous study [6], we measured 2,3,7,8-substituted PCDD/Fs and dl-PCBs in whole blood samples after freeze-drying samples in an EYELA freeze dryer (FDU-1200; Tokyorika Inc., Tokyo, Japan) to extract fat using an ASE-200 accelerated solvent extractor (Dionex Corporation, Sunnyvale, CA, USA). Then, internal standards 13C-labeled for PCDDs/Fs and dl-PCBs were added to the samples. After purification and separation by chromatography, the PCDD/F and non-ortho PCB fraction was collected and 17 PCDD/F congeners and 4 non-ortho PCB congeners were quantified using a gas chromatograph (HP-6980; Hewlett-Packard, Palo Alto, CA, USA) equipped with a high-resolution mass spectrometer (MStation-JMS700; JEOL, Tokyo, Japan). Further details of the pretreatment process have been reported previously [7,22]. The TEQ values of PCDD/Fs and non-ortho PCBs in each sample were calculated by multiplying each congener concentration by its TEF from the WHO 2005 TEF list [33] and expressed as the lipid base, as well as the concentrations of all congeners. For TEQ-dl-PCBs, only two congeners of non-ortho PCBs, TCB#77 and HxCB#169, were included because the levels of TCB#81 and PeCB#126 congeners for almost all samples were lower than the detection limits.

### 2.4. Statistical Analysis

We performed statistical analysis using SPSS version 22.0 (IBM; Armonk, NY, USA). The BEQ levels were compared between the exposed and unexposed groups or among the three different residency groups after adjusting for confounding factors including age, serum cholesterol levels, and smoking habit using a general linear model. The levels of WHO-TEQ and BEQ were also compared between shorter- and longer-residency groups after adjusting for confounding factors using a general linear model.

Associations between the levels of TCDD and five dioxin TEQ and BEQ levels were analyzed using Spearman’s correlations (ρ) and a linear regression model after adjusting for age, serum cholesterol, and smoking habit. Spearman’s ρ and the linear regression model, after adjusting for serum lipid levels, were also used to analyze associations between the fat contents of bioassay samples and the levels of dioxin exposure markers.

For all tests, *p* < 0.05 was considered significant.

## 3. Results

### 3.1. Comparisons of Adjusted BEQ Levels Between Exposed and Unexposed Men to Dioxins

For both crude and fat-based BEQ levels, the adjusted means in exposed men in Bien Hoa were significantly higher than those in unexposed men in Hanoi (Table 2). The exposed/unexposed ratio of the adjusted means was 2.3 for crude BEQ and 2.7 for fat-based BEQ, suggesting that the difference between exposed and unexposed men might be greater for fat-based BEQ.

### 3.2. Correlations Between BEQ and WHO-TEQ Levels in 32 Men in Bien Hoa

To investigate the correspondence with WHO-TEQ levels measured by an instrument, associations between the levels of TCDD and five dioxin TEQs (fat-based values) and fat-based BEQ levels were analyzed, as well as crude BEQ levels, in 32 men in Bien Hoa. It is worth noting that fat correction might not be suitable in the case of very low amounts of serum lipids due to small sample volumes. Spearman’s ρ (unadjusted correlation) and the standardized regression coefficient β after adjusting for age, serum cholesterol, and smoking habit are shown in Table 3.

Crude BEQ levels significantly correlated (Spearman’s ρ) with TEQ-dl-PCBs and TEQ-PCDD/Fs/dl-PCBs only, but the standardized β values adjusted for age, serum cholesterol, and smoking habit were significant between the crude BEQ levels and TCDD, TEQ-PCDDs, TEQ-PCDD/Fs, and PCDD/Fs/dl-PCBs, suggesting that serum lipid was a confounding factor. By contrast, fat-based BEQ levels were significantly correlated with TCDD, TEQ-PCDDs, TEQ-PCDD/Fs, and TEQ-PCDD/Fs/dl-PCBs, with (β values) and without (ρ values) adjustment for confounding factors. Compared with crude BEQ levels, the β values of these dioxin exposure markers were higher for fat-based BEQ levels, suggesting that fat-based BEQ is a better indicator of WHO-TEQs than crude BEQ.

**Figure 3:**
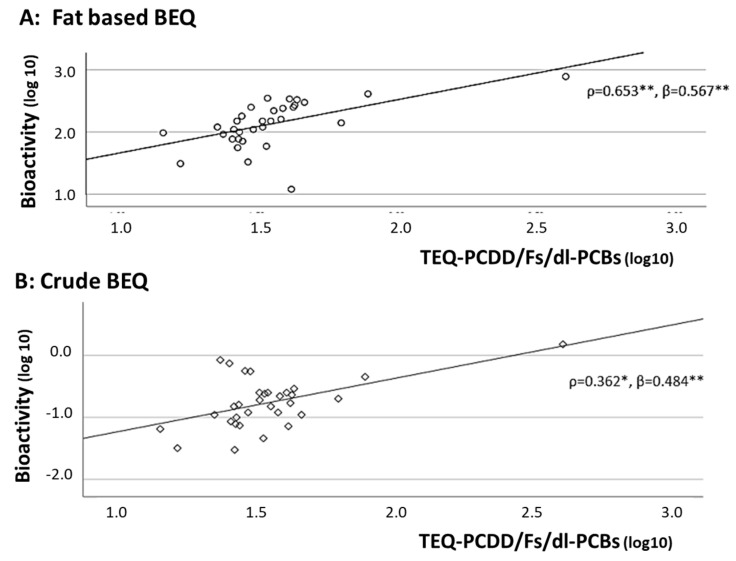
Scatter plots between TEQ−PCDD/Fs/dl−PCBs levels by instrumental analysis and fat-based (**A**) and crude (**B**) BEQ levels. log10: logarithm base 10, *: *p* < 0.05, **: *p* < 0.01, ρ: Spearman’s correlation coefficients, β: standardized regression coefficients after adjusting for age, serum cholesterol, and smoking (yes, no).

In addition, scatter plots between crude and fat-based BEQ levels and TEQ-PCDD/Fs/dl-PCBs, using the log10-transformed values, are shown in Figure 3, indicating their distributions and fitness of the regression lines between TEQ-PCDD/Fs/dl-PCB levels and fat-based levels (A) or crude levels (B). Fitness was higher for fat-based BEQ levels, with one exception.

### 3.3. Comparisons of Adjusted BEQ Levels of Exposed Men with Shorter and Longer Residency in Bien Hoa Compared with Unexposed Men in Hanoi

To confirm the higher BEQ levels of exposed men in Bien Hoa with shorter and longer residency compared with unexposed men in Hanoi, three exposure groups in total were compared after adjusting for age, serum cholesterol, and smoking habit using the ANCOVA model. The test results for the shorter- or longer-residency groups compared with the unexposed reference group are shown in Table 4.

For crude BEQ levels, the adjusted mean level was significantly higher in Bien Hoa men with shorter residency (*p* = 0.001), but no significant difference was found between Bien Hoa men with longer residency and unexposed men in Hanoi (*p* = 0.096). By contrast, fat-based BEQ levels in both men with shorter and longer residency were significantly higher than unexposed men (*p* < 0.001, and *p* = 0.034, respectively). The adjusted means of fat-based BEQ levels were three times higher in the shorter-residency group and two times higher in the longer-residency group, compared with that in the unexposed group, although there was no significant difference in the BEQ levels between the shorter- and longer-residency groups. These results suggest that fat-based BEQ is a more useful biomarker compared with crude BEQ to distinguish dioxin-exposed from unexposed individuals.

### 3.4. Comparisons of WHO-TEQ and BEQ Levels Between Men with Shorter and Longer Residency in Bien Hoa

Although there was no significant difference in the adjusted BEQ levels between the two residency groups, the adjusted mean of fat-based BEQ of the shorter-residency group was 1.6 times higher (161 vs. 104) than that of the longer-residency group in Bien Hoa (Table 4). To investigate which dioxin congener contributes to the difference in fat-corrected BEQ levels between the two groups, the TCDD concentration and five dioxin TEQ levels were compared between the groups after adjusting for age. The results, including the shorter/longer-residency group ratio for each dioxin marker, are shown in Table 5.

No significant difference was found between the shorter- and longer-residency groups for any dioxin marker, but the shorter/longer-residency group ratio was 1.9 for TCDD, which was the highest of all dioxin markers, followed by 1.7 for TEQ-PCDD and 1.5 for TEQ-PCDD/F and TEQ-PCDD/F/dl-PCB. These results indicated that PCDD congeners, particularly TCDD, may contribute to the increased fat-based BEQ level in the shorter-residency group in Bien Hoa.

### 3.5. Fat Contents of Bioassay Samples and Dioxin Exposure

Finally, we investigated the influence of dioxin exposure on the fat content of the samples, resulting in alterations in the fat-based BEQ levels. The fat content of 1 g of sample for the shorter- and longer-residency groups in Bien Hoa was significantly lower than for the unexposed Hanoi men, indicating that the increased fat-based BEQ level was due to the lower fat content of the samples from men in Bien Hoa (Table 1). Next, for the 32 men in Bien Hoa, we investigated the associations between blood levels of TCDD and five TEQs and the fat content of the bioassay samples by a regression model after adjusting for serum cholesterol and log10-transformed triglyceride levels (Table 6). Only TEQ-dl-PCB was significantly correlated (Spearman’s ρ) with fat content. However, none of the dioxin markers showed significantly increased/decreased standardized β values after adjusting for serum fat levels, suggesting that factors other than dioxin exposure may be related to the lower fat levels in men in Bien Hoa.

## 4. Discussion

### 4.1. Fat BEQ and WHO-TEQ Levels, as Measured by an Instrument, in Dioxin-Exposed Men

In the present study of Vietnamese middle-aged men, the fat-based BEQ levels were nearly three times higher and the adjusted crude BEQ levels were two times higher in exposed Bien Hoa men compared with unexposed Hanoi men. Significant correlations with dioxin TEQs, as measured by instrumental analysis, were detected for both crude and fat-based BEQ levels, but higher correlations were found for fat-based BEQ levels. These results suggested that fat-based BEQ levels may be better dioxin exposure markers than crude BEQ levels, even if the amounts of serum lipids for fat-correction are very low due to small sample volumes. Moreover, the fat-based BEQ levels in Bien Hoa men with longer residency but comparatively lower WHO-TEQ levels were significantly higher than those in unexposed men, as well as those in Bien Hoa men with shorter residency but higher WHO-TEQs. This suggests that fat-based BEQ, as determined by the DR-EcoScreen bioassay, is a significant exposure marker to distinguish dioxin-exposed men from unexposed men.

In our previous study targeting the same subjects in Bien Hoa [22], we reported that blood WHO-TEQ levels were significantly higher in men who worked in the airbase or in those who used herbicides and pesticides, suggesting that higher BEQ levels in men with shorter residency may be caused by occupational dioxin exposure related with the Bien Hoa airbase or herbicide use for their farm work. Furthermore, among these subjects in Bien Hoa, the rate of suspected exposure to dioxins during the perinatal period was 90% among men with longer residency, compared with 13% among men with shorter residency, suggesting that the longer-residency group may have experienced dioxin impacts on brain development associated with perinatal exposure [34], although the BEQ levels were similar between the two groups. In future studies, we intend to investigate the difference in brain regional volumes not only between the shorter- and longer-residency groups but also in the unexposed group.

In the present study, we found that the ratio of fat-based BEQ levels between the shorter/longer-residency groups was 1.6, whereas the ratio was 1.5 for TEQ-PCDD/Fs or TEQ-PCDD/Fs/dl-PCBs, suggesting increased AhR activity in the shorter-residency group. Because the ratios of TCDD and TEQ-PCDDs were 1.9 and 1.7, respectively, it was suggested that higher levels of PCDD congeners, particularly TCDD, may contribute to the increased BEQ ratio and higher level of AhR activity in the shorter-residency group than in the longer-residency group.

Taken together, fat-based BEQ, as determined by the DR-EcoScreen assay, seems to be an effective measure of dioxin exposure levels, as estimated by the WHO-TEQ, and may be useful for exposure assessment in epidemiological studies in Vietnam.

### 4.2. Factors Relevant to BEQ Levels in Previous Epidemiological Studies

In the FLEHS II study, Cross et al. (2011) [28] reported a difference of more than three times in the BEQ levels correlating to sex (boys > girls), as measured by a third-generation CALUX bioassay using the H1L7.5c1 hepatoma cell line and serum samples from adolescents in Belgium. Because the current study only targeted men, we were not able to investigate sex differences in the BEQ levels. In future studies, it would be interesting to investigate the BEQ levels of adolescents from our birth cohorts, including subjects of both sexes without job-related dioxin exposure. Job-related dioxin exposure was associated with increased dioxin levels in blood in our previous study on men in Bien Hoa [22].

In Greenland, a mother–child cohort termed ACCEPT (Adapting to Climate Change, Environmental Pollution and Dietary Transition) was established in 2010–2015 to evaluate maternal exposure levels to persistent organic pollutants (POPs) during pregnancy by assessing dioxin-like activity using an AhR transactivation assay with the mouse hepatoma cell line Hepa1.12cR, and investigated their possible association with fetal development and infant/child health [36]. Previous studies by this group showed that smoking status influenced serum POP-induced dioxin-like activity, and serum lipids related to seafood intake and lifestyle were adjusted for as an important confounding factor [36,37,38].

Therefore, in the current study, we analyzed the relationships between BEQ levels in the different groups according to the location or length of residency, including no residence in exposed areas, after adjusting for age, serum cholesterol levels, and smoking habit. Particularly, serum cholesterol levels were supposed to be a powerful confounding factor because of significantly lower levels in both exposed men in Bien Hoa compared with unexposed men in Hanoi. However, in the statistic analytical model, the statistical effect of serum cholesterol was not significant, whereas age was significantly associated with BEQ levels.

### 4.3. Serum Sample Measurement Using a Bioassay in Countries Other than Vietnam

Warner et al. (2005) [27] measured dioxins and PCBs in serum by the instrumental method and calculated total TEQ levels in 78 women living in an area near Seveso, Italy, where accidental exposure to TCDD at extremely high levels occurred as a result of an industrial explosion. They also measured 32 samples by a CALUX bioassay and investigated correlations between the samples. However, the BEQ levels of 10 samples from the 32 selected samples were under the LOD. In addition, no significant correlation was found between the BEQ levels and total TEQ levels. For these CALUX analyses, the authors used the original CALUX bioassay with the H1L6.1c3 mouse cell line, which lacked sufficient sensitivity.

In the FLEHS II study, 45 pooled samples from among 173 serum samples were measured by both instrumental analysis and the improved CALUX bioassay, followed by BEQ measurements of all participants [28]. For the pooled samples, the BEQ/TEQ ratio was 2.0 for PCDD/Fs, indicating that the BEQ levels were nearly two times higher than the WHO-TEQ levels for PCDD/Fs. However, for our samples in Bien Hoa (n = 32), the BEQ/TEQ ratios were 4.0 for PCDD/Fs and 3.8 for PCDD/Fs/dl-PCBs (BEQ = 134 pg-BEQ/g-fat, PCDD/Fs = 33.7 pg-TEQ/g-fat, PCDD/Fs/dl-PCBs = 35.4 pg-TEQ/g-fat), suggesting that the biological activity detected by the DR-EcoScreen bioassay was higher than that detected by the CALUX bioassay used in the FLEHS II study [28]. This indicates that the DR-EcoScreen bioassay has higher sensitivity for AhR detection. Furthermore, the higher contribution of PCDD congeners, particularly TCDD, among the Bien Hoa men in the current study might be another reason for the higher biological activity of AhR (as discussed in Section 4.1).

Budin et al. (2021) [39] determined the relative potency (RP) values of PCDD/F congeners using the DRhuman CALUX assay and suggested that the WHO-TEFs may be underestimated for some PCDD/F congeners, particularly for 1,2,3,4,7,8-HxCDD and HpCDD, which had RP values 20 and 40 times higher than those suggested by the WHO-TEFs, respectively. To verify their findings, we recalculated the TEQ-PCDDs and TEQ-PCDD/Fs values of our Bien Hoa samples using their RP values and analyzed the correlations between the fat-based BEQ levels and the recalculated TEQ levels. Although the recalculated TEQ-PCDDs and TEQ-PCDD/Fs were significantly correlated with fat-based BEQ levels, as well as WHO-TEQs, the correlation coefficients and standardized β-values were lower for the recalculated TEQs compared with the WHO-TEQs for our Bien Hoa samples. Furthermore, the ratio of the shorter/longer-residency groups was 0.9 for the recalculated TEQ-PCDD/Fs, compared with 1.6 for the fat-based BEQ, suggesting that the TEQ levels based on the WHO-TEFs may be consistent with the BEQ levels determined by the DR-EcoScreen bioassay. Taken together, the DR-EcoScreen bioassay may be a useful measure of AhR activity in Vietnamese samples typically exposed to TCDD originating from Agent Orange.

In mothers from the ACCEPT cohort, who were mainly exposed to lipophilic POPs and PCBs, the mean BEQ was 86.2 (pg/g lipid) [36]. PCB156 was suggested to contribute to the increasing BEQ levels (an increase of 1 g/L PCB156 relates to an increase of 6.59 pg-BEQ/mL) statistically analyzed by principal component analysis. However, the total dl-PCBs or dl-PCBs relative to the total PCBs did not significantly contribute to the increasing BEQ levels, suggesting that further methodological improvement of the bioassay might be necessary.

## 5. Conclusions

The results of this study suggest that fat-based BEQ is a more effective marker of dioxin-like activity than crude BEQ to distinguish dioxin-exposed men from unexposed men in Vietnam. Higher levels of PCDD congeners, particularly TCDD, whose WHO-TEQ values are higher than those of other congeners, may contribute more to increased BEQ levels. This suggests that the DR-EcoScreen bioassay is a useful measure to analyze dioxin-like activity associated with dioxin exposure of residents in a hotspot of dioxin contamination originating from Agent Orange in Vietnam.

## Figures and Tables

**Table 1:** Comparisons of characteristics’ comparisons between exposed men with shorter and longer residency in Bien Hoa and unexposed men in Hanoi.

Residency	Shorter Residency in Bien Hoa		Longer Residency in Bien Hoa		Residency in Unexposed Hanoi		*p*-Val. 1	*p*-Val. 2
	(N = 22)		(N = 10)		(N = 32)			
	Mean	SD	Mean	SD	Mean	SD		
Length of residency (years)	13.9	6.7	38.1	4.5	-	-		
Age (years)	34.1	5.7	38.3	5.4	39.3	4.2	0.001	0.841
Education (years)	12.2	3.3	11.5	3.0	13.4	3.2	0.385	0.256
Height (cm)	164.9	4.7	166.5	5.7	165.6	5.2	0.795	0.859
Weight (kg)	64.0	9.2	71.9	11.2	64.0	8.6	0.988	0.055
BMI	23.5	3.1	25.9	3.4	23.0	2.4	0.977	0.035
% fat	24.0	3.1	24.8	2.7	22.3	7.4	0.856	0.663
Serum cholesterol (mmol/L)	4.7	0.8	4.8	0.8	5.6	1.1	0.003	0.046
Serum triglyceride (mmol/L) *	2.2	2.1	1.6	1.8	2.6	2.0	0.512	0.126
Assay samples								
Sample volume (g)	1.00	0.05	0.99	0.06	0.96	0.10	0.043	0.313
Fat mass in a sample (mg)	5.2	2.6	4.5	0.8	8.1	1.8	0.001	0.001
Fat content (mg/g sample)	5.2	2.5	4.5	0.7	8.5	1.8	0.001	0.001

N: number of subjects, SD: standard deviation; BMI: body mass index; *: geometrical mean, *p*-val. 1: *p*-value for men with shorter residency (<30 years) vs. unexposed men, *p*-val. 2: *p*-value for men with longer residency (≥30 years) vs. unexposed men.

**Table 2:** Comparisons of adjusted crude and fat-based BEQ levels between dioxin exposed men in Bien Hoa (N = 32) and unexposed men in Hanoi (N = 32).

Bioactivity Index	Residency	Mean	SD	Adj Mean	SE	95%CI		*p*-Value
Crude BEQ	Unexposed Hanoi	0.09	1.5	0.08	1.2	0.06	0.11	ref.
	Exposed Bien Hoa	0.16	2.8	0.18	1.2	0.14	0.25	<0.001
Fat-based BEQ	Unexposed Hanoi	53	1.8	51	1.2	39	68	ref.
	Exposed Bien Hoa	134	2.3	138	1.2	105	183	<0.001

N: number of subjects, SD: standard deviation; Adj Mean: mean adjusted for age, serum cholesterol, smoking (yes, no); SE: standard error; CI: confidence interval, ref.: reference.

**Table 3:** Associations between the BEQ levels and dioxin WHO-TEQs measured by instrument analysis in Bien Hoa men (N = 32).

	Crude BEQ (/g Weight)		Fat-Based BEQ (/g Fat)	
	ρ	β	ρ	β
TCDD	0.274	0.404 *	0.561 **	0.511 **
TEQ-PCDDs	0.305	0.470 **	0.627 **	0.568 **
TEQ-PCDFs	0.130	0.039	0.217	0.088
TEQ-PCDD/Fs	0.336	0.477 **	0.627 **	0.568 **
TEQ-dl-PCBs	0.549 **	0.201	0.349	−0.062
TEQ-PCDD/F/dl-PCBs	0.362 *	0.484 **	0.653 **	0.567 **

*: *p* < 0.05, **: *p* < 0.01, ρ: Spearman’s correlation coefficients, β: standardized regression coefficients after adjustment for age, serum cholesterol, and smoking (yes, no).

**Table 4:** Comparisons of adjusted crude and fat-based BEQ levels among three exposure groups including dioxin exposed men in Bien Hoa with shorter residency or longer residency, and unexposed men in Hanoi.

Bioactivity Index	Residency	N	Mean	SD	Adj Mean	SE	95%CI		*p*-Value
Crude BEQ	Unexposed Hanoi	32	0.09	1.5	0.08	1.2	0.06	0.11	ref.
	Shorter residency in Bien Hoa	22	0.17	2.8	0.21	1.2	0.15	0.30	0.001
	Longer residency in Bien Hoa	10	0.15	3.0	0.15	1.3	0.09	0.24	0.096
Fat-based BEQ	Unexposed Hanoi	32	53	1.8	51	1.1	38	67	ref.
	Shorter residency in Bien Hoa	22	149	2.5	161	1.2	115	227	<0.001
	Longer residency in Bien Hoa	10	106	2.1	104	1.3	65	166	0.034

N: number of subjects, SD: standard deviation; Adj Mean: mean adjusted for age, serum cholesterol, smoking (yes, no); SE: standard error; CI: confidence interval, ref: reference.

**Table 5:** Comparisons of seven dioxin exposure marker levels, TCDD, five WHO-TEQs and fat-based BEQ levels, between men with shorter and longer residency in Bien Hoa.

	Shorter Residency (N = 22)				Longer Residency (N = 10)				*p*-Val.	Ratios
Dioxins in Blood	Mean	SD	Adj Mean	SE	Mean	SD	Adj Mean	SE		
TCDD	8.3	3.1	9.0	1.3	5.6	2.0	4.8	1.4	0.157	1.9
TEQ-PCDDs	26.8	2.1	28.4	1.2	19.0	1.5	16.8	1.2	0.060	1.7
TEQ-PCDFs	8.5	1.3	8.6	1.1	8.7	1.2	8.5	1.1	0.894	1.0
TEQ-PCDD/Fs	36.4	1.9	38.2	1.1	28.3	1.3	25.4	1.2	0.089	1.5
TEQ-dl-PCBs	1.1	4.9	1.2	1.3	1.3	1.8	1.0	1.5	0.744	1.2
TEQ-PCDD/F/dl-PCBs	38.4	1.9	40.4	1.1	29.7	1.3	26.5	1.2	0.073	1.5
Fat-based BEQ	149	2.5	161	1.2	106	2.1	104	1.3	0.299	1.6

N: number of subjects, SD: standard deviation; Adj Mean: mean adjusted for age, serum cholesterol, smoking (yes, no); SE: standard error; *p*-val.: *p*-value, Ratios: ratios of adjusted means between shorter- and longer-residency groups.

**Table 6:** Associations between fat contents of bioassay samples and levels of dioxin exposure markers and serum lipids (cholesterol and triglyceride).

	ρ	β
Dioxin exposure markers		
TCDD	0.017	0.150
TEQ-PCDDs	−0.066	0.139
TEQ-PCDFs	0.024	0.066
TEQ-PCDD/Fs	−0.006	0.163
TEQ-dlPCBs ^#^	0.496 **	0.162
TEQ-PCDD/F/dlPCBs	0.041	0.184
Serum lipids		
Cholesterol	0.331	-
Triglyceride	0.623 **	-

**: *p* < 0.01, #: one of the confounding factors for β: standardized correlation coefficients.

## Data Availability

The data presented in this study are available on request to the corresponding author. The data are not publicly available due to privacy or ethical restrictions.
